# BCAR1 plays critical roles in the formation and immunoevasion of invasive circulating tumor cells in lung adenocarcinoma

**DOI:** 10.7150/ijbs.61790

**Published:** 2021-06-11

**Authors:** Chun-Guo Mao, Sha-sha Jiang, Xiao-yang Wang, Shao-Lin Tao, Bin Jiang, Cheng-Yi Mao, Yan-Lian Yang, Zhi-Yuan Hu, Tan Long, Hua Jin, Qun-You Tan, Yi Huang, Bo Deng

**Affiliations:** 1 Thoracic Surgery Department, Institute of Surgery Research, Daping Hospital, Army Medical University, Chongqing 400042, China.; 2 Biomedical Analysis Center, Army Medical University, Chongqing 400038, China.; 3 Department of Pathology, Daping Hospital, Army Medical University, Chongqing 400042, China.; 4 CAS Key Laboratory of Standardization and Measurement for Nanotechnology, CAS Key Laboratory for Biomedical Effects of Nanomaterials and Nanosafety, CAS Center for Excellence in Nanoscience, National Center for Nanoscience and Technology of China, Beijing 100190, China.; 5 School of Nanoscience and Technology, Sino-Danish College, University of Chinese Academy of Sciences, Beijing 100049, P. R. China.

**Keywords:** Breast cancer anti-estrogen resistance 1 (BCAR1/p130Cas), lung cancer, circulating tumor cells, prognosis, migration, invasion, Epithelial-to-mesenchymal transition, anoikis, CD274, RAC1, BRD4, exosome

## Abstract

**Background:** We investigated the roles of breast cancer anti-estrogen resistance 1 (BCAR1/p130Cas) in the formation and immunoevasion of invasive circulating tumor cells (CTCs) in lung adenocarcinoma (LUAD).

**Methods:** Biomarkers of CTCs including BCAR1 and CD274, were evaluated by the CanPatrol method. Proteomics analysis of LUAD cells and exosomes after BCAR1 overexpression (BCAR1-OE) was performed by mass spectrometry. Cell functions and relevant signaling pathways were investigated after BCAR1 knockdown (BCAR1-KO) or BCAR1-OE in LUAD cells. Lastly, *in vitro* and *in vivo* experiments were performed to confirm the roles of BCAR1 in the formation and immunoevasion of CTCs.

**Results*:***High expression of BCAR1 by CTCs correlated with CD274 expression and epithelial-to-mesenchymal transition (EMT). RAC1, together with BCAR1, was found to play an important role in the carcinogenesis of LUAD. RAC1 functioned with BCAR1 to induce EMT and to enhance cell proliferation, colony formation, cell invasion and migration, and anoikis resistance in LUAD cells. BCAR1 up-regulated CD274 expression probably by shuttling the short isoform of BRD4 (BRD4-S) into the nucleus. CTCs, as well as tumor formation, were prohibited in nude mice xenografted with BCAR1-KO cells. The co-expression of BCAR1/RAC1 and BCAR1/CD274 was confirmed in LUAD. BCAR1 expression in LUAD is an indicator of poor prognosis, and it associates with immunoevasion.

**Conclusion*:***BCAR1, as a new target for the treatment of LUAD, plays roles in the formation and immunoevasion of invasive CTCs. The mechanism includes triggering EMT via RAC1 signaling and up-regulating CD274 expression by shuttling BRD4-S into the nucleus.

## Introduction

Lung adenocarcinoma (LUAD), the most frequently diagnosed cancer and the leading cause of cancer death, accounts for 11.6% of newly diagnosed cancer cases and 18.4% of cancer deaths 1, with 5-year stage-specific survival rates ranging from 73% for stage IA disease to 13% for stage IV disease 2.

Circulating tumor cells (CTCs) are cancer cells released from the primary tumor or metastatic sites that circulate in the bloodstream, with the ability to invade and colonize a distal site and encourage tumor growth 3. However, the mechanism responsible for the formation and immunoevasion of CTCs remains unclear.

Breast cancer anti-estrogen resistance 1 (BCAR1/p130Cas) is a scaffold protein serving as a node in signaling pathways and participating in the signal transduction of several oncogenic kinases e.g., the Abelson (ABL) and focal adhesion kinase (FAK) 4. BCAR1 was reported to be a general regulator of oncogenic-mediated cancer cell growth and invasiveness.

BCAR1 is overexpressed in a variety of malignancies, e.g., cancers of the breast, lung, liver, and brain. It also promotes invasion and metastasis of these primary tumors 5. Previously, we reported that BCAR1 expression in LUAD promotes epithelial-to-mesenchymal transition (EMT) 6, illustrating the importance of BCAR1 in carcinogenesis 7,8. Intriguingly, BCAR1 was detected in CTCs, indicating that BCAR1 is involved in LUAD progression 9.

Here, we performed *in vivo* and *in vitro* studies to understand the clinical and translational significance of BCAR1 in CTCs in LUAD.

## Patients and Methods

### Patients and clinical databases for validation

The study protocol was approved by the Research Ethics Board of Daping hospital (Chongqing City, P.R. China) [IRB: 2018-083], Written informed consent was obtained from all participants. All cases underwent surgery, and pulmonary neoplasm was confirmed by pathology. None of the cases received treatment before enrollment in the study. The postoperative follow-up was conducted via telephone or mail.

We enrolled patients with early stage LUAD due to fewer potential confounders in the analysis of survival. Additional inclusion criteria were as follows: (1) no anti-tumor therapy prior to surgery; (2) no history of chronic diseases, (e.g., COPD, hepatitis and tuberculosis); (3) >18 years of age; and (4) Eastern Cooperative Oncology Group (ECOG) performance status score of 0 or 1.

Eighty cases from January 2016 to April 2019 were enrolled to evaluate BCAR1 expression in CTCs prior to surgery and disease-free survival (DFS) rather than overall survival (OS), due to inadequate follow-up time for OS evaluation (Table S1). Twenty cases from January 2019 to May 2020 were enrolled to evaluate BCAR1 and CD274 expression in CTCs. Twenty one cases from January 2019 to May 2020 were enrolled to evaluated BCAR1 expression in CTCs isolated by the CytoploRare method as a validation.

Forty patients from July 2005 to December 2009 were recruited as a training cohort to evaluate BCAR1 expression in tumor tissues and OS. In the aforementioned 80 cases, there were 54 patients with sufficient LUAD tissues to evaluate the protein expression.

Clinical demographic features of the cohort are listed in Table S1. Ten patients with benign lung tumors and 31 healthy individuals served as controls. Subjects with bronchiectasis, viral hepatitis, ischemic cardiac disease or cerebrovascular disease were excluded.

Genes expressions (RNA-seq V2) in LUAD tissues were retrieved from TCGA data using the GEPIA Tool (http://gepia.cancer-pku.cn) 10. We used log2 (normalized count) of mRNAs expressions for the analysis. Furthermore, immune scores were calculated using the ESTIMATE algorithm in LUAD tissues retrieved from TCGA data 11. The TIMER 2.0 Program was used for analysis of tumor-infiltrating immune cells 12.

### Preparation of tissue microarrays and immunohistochemical analysis

Tissue microarrays of training and study cohorts were prepared as previously described 13. Immunohistochemistry was performed using the antibodies listed in Table S2. To evaluate expression, the mean integrated optical density was determined with Image-Pro Plus 6.0 Software (Media Cybernetics, Inc., USA). CD8+ and CD4+ T cells were counted with iViewer Software (Unic Technologies, China), and the number of T cells in five points within the same area was counted and averaged.

### Detection of BCAR1 expression in CTCs

#### Detection of BCAR1 expression in CTCs using the CanPatrol method

As previously described 14, blood (~10 mL) was collected from participants 1-2 days before surgery. The CanPatrol method was used to detect CTCs and relative biomarkers 15. In brief, erythrocytes were removed by red blood cell lysis and CD45+ leukocytes were depleted in blood samples using magnetic beads. CTCs were enriched using 8-μm pore size calibrated membrane filters. CTCs were identified and characterized using RNA *in situ* hybridization, which is based on the branched DNA signal amplification technology. DAPI(+)/CD45(-) cells were defined as CTCs^15^. EMT markers, (e.g., cytokeratins 8, 18, and 19, epithelial cell adhesion molecule, vimentin, and twist) were detected as previously described 15. BCAR1 and CD274 primers, which were used to prepare probes are listed in Table S3.

#### Detection of BCAR1 in CTCs using the CytoploRare method

CTCs were evaluated using the CytoploRare method (GenoSaber Biotech Co., Ltd., China) 16, 17. Blood (~3 ml) was collected from participants 1-2 days before surgery. Erythrocytes were removed by red blood cell lysis, and CD45+ leukocytes were depleted using magnetic beads 16, 17. CTCs were stained with the BCAR1 antibody (Table S2) and analyzed.

### BCAR1-knockout (KO) in H1975 and H1299 cells and BCAR1-overexpression (OE) in A549 cells

#### BCAR1-KO in H1975 and H1299 cells

BCAR1-KO in H1975 and H1299 was conducted using CRISPR/Cas9 technology. Firstly, the lenti-Cas9-puro vector (Jikai Gene Co., Ltd., China, Fig. S1A) was transfected into H1975 and H1299 cells. H1975-Cas9 and H1299-Cas9 cells were stably screened with puromycin (2ug/ml). Secondly, the sgRNA of BCAR1 as an interference target sequence (GenBank NM_001170714) and the paired negative control sgRNA were designed (Table S4). Primers were annealed to form double-stranded DNA and linked to the digested sgRNA vector, i.e., GV371 (Jikai Gene Co., Ltd., China, Fig. S1A). Positive plasmids were obtained and identified by sequencing. Finally, lentiviral plasmids containing sgRNA were transfected into H1975-Cas9 and H1299-Cas9 stable cell lines, respectively, which were monitored by fluorescence. KO efficacy was evaluated by western blotting. Grayscale values of target proteins were analyzed with Image J 1.52 software (National Institutes of Health, USA).

#### BCAR1-OE in A549 cells

Primers used for the preparation of the cDNA library are listed in Table S5. Target fragments were amplified by PCR and inserted into the CV146 vector (Jikai Gene Co., Ltd., China, Fig. S1A). The construct was verified by sequencing, followed by transfection into A549 cells. The empty vector served as the negative control. A549 BCAR1-OE cells were screened with puromycin (2 µg /mL). OE efficacy was evaluated by q-PCR and western-blotting.

#### Measurement of BCAR 1, RAC1, BRD4, CD274 and EMT biomarker expression

Protein lysates were used for western blotting. The antibodies are listed in Table S2. The BCAR1 and CD274 primers used for q-PCR are listed in Tables S6 and S7, respectively.

#### Protein interaction assay and extraction of nucleoproteins

GV367 (Jikai Gene Co., Ltd., China, Fig. S1A) and FLAG-RAC1 primers (Table S8) were used to prepare vectors, which were transfected into A549 cells for Co-IP to detect the interaction between RAC1 and BCAR1 using the antibodies listed in Table S2. Co-IP was used to evaluate the interaction between BCAR1 and BRD4 using ANTI-FLAG M2 Affinity Gel (#A2220, Sigma USA) according to the manufacturer's instructions. Nucleoproteins were extracted using the Nuclear and Cytoplasmic Protein Extraction Kit (#P0027, Beyotime, China) according to the manufacturer's instructions.

#### Treatment with RAC1 inhibitor and detection of active-RAC1

Cells were treated with NSC 23766, a RAC1 inhibitor (APExBIO, USA) 18 at 10 µM or 50 µM for 48h. Active-RAC1 was detected using the Active RAC1 Detection Kit (#8815, CST, USA) according to the manufacturer's instructions. In brief, A549-BCAR1-OE cell lysates were incubated with GTPrs or GDP to activate or inactivate RAC1, respectively, and GST-PAK1-PBD was used to activate GTP-bound RAC1, which was measured by western blotting.

### Detection of cell functions

Cell proliferation was assessed using the MTT assay according to the manufacturer's protocol 19. Colony formation was determined as previously described 20. Cell invasion and migration were assessed using the Transwell Assay (#3422; Corning, USA) as previously described 7. Cell anoikis was determined using the CytoSelect Anoikis Assay (#CBA-080, Cell Biolabs, USA) as previously described 21.

### Isolation and characterization of exosomes

#### Isolation of exosomes by ultracentrifugation

Conditioned cell media were collected, followed by centrifugation at 800 *g* for 5 min and 12000 *g* for 20 min at 4 °C. Supernatants were filtered through a 0.22-μm pore filter (Millipore, USA) and ultracentrifuged (Himac CP-80WX, Hitachi, Japan) at 110000 *g* for 3 h at 4 °C. Pellets were collected, resuspended in HBSS, and ultracentrifuged at 110000 *g* for 2 h at 4 °C. Pellets were resuspended in HBSS or RIPA buffer and stored at -80 °C until use.

#### Nanoparticle tracking analysis of exosomes

Exosomes (30 μL) resuspended in HBSS, were diluted with PBS (Biological Industries, Israel). The exosome size and concentration were measured using the ZetaView PMX 110 System and ZetaView 8.04.02 Software (Particle Metrix, Germany), which involve nanoparticle tracking analysis (NTA). Eleven positions were pre-set for NTA, and the system was calibrated using 110-nm polystyrene particles. The temperature was maintained between 25 °C and 27 °C.

#### Transmission electron microscopy (TEM)

Exosomes (10 μL) resuspended in HBSS, were applied to 200 -mesh carbon-coated copper grids for 10 min at 24 °C and negatively stained with phosphotungstic acid (pH 6.5). Images were acquired with a JEM-1400 transmission electron microscope (JEOL, Japan).

### Label-free LC-mass spectrometry (MS) quantitative analysis of proteins

#### Proteomics analysis of exosomes

Exosomes resuspended in RIPA buffer were centrifuged at 10000 *g* for 5 min, and supernatants were collected for determination of the protein concentration using the Bradford assay (ThermoFisher Scientific, USA).

Equivalent amounts of protein (60 µg) were digested and analyzed by nanoElute UHPLC (Bruker Daltonics, Germany) coupled to the timsTOF Pro System (Bruker Daltonics) which was equipped with a CaptiveSpray ion source. Peptides (300 ng) were separated on a 25-cm analytical column with a packed emitter tip (75 μm ID, 1.6 μm C18 beads, Aurora Series with CSI, IonOpticks, Australia). The column temperature was maintained at 50 °C using an integrated Toaster column oven. Mobile phases A and B were water and acetonitrile containing 0.1 vol% formic acid, respectively. Mobile phase B was linearly increased from 2 to 22% within 90 min, followed by an increase to 37% within 10 min and a further increase to 80% within 10 min , which was sustained for 10min (total separation method time, 120 min). The timsTOF Pro System was operated in positive ion data dependent acquisition PASEF mode using Compass Hystar 5.1.8.1 Software. Settings were as follows: mass range 100 to 1700 m/z; 1/K0 start 0.75 Vs/cm^2^ end 1.4 Vs/cm^2^; ramp time 100ms, lock duty cycle 100%; capillary voltage 1400V; dry gas 3 L/min; and dry temp 180 °C, The PASEF settings were as follows: 10 MS/MS scans (total cycle time 1.16 sec); charge range 0-5; active exclusion for 0.4 min; scheduling target intensity 1 X 10^4^; and intensity threshold 2500. The collision energy was ramped linearly as a function of the mobility from 59 eV at 1/K0=1.6 Vs/cm^2^ to 20 eV at 1/K0=0.6 Vs/cm^2^.

Raw MS data were submitted to the Peaks Studio X Pro Software for protein identification and label free quantitation (LFQ). Data were searched against the SwissProt database of 20420 human sequences with trypsin as the enzyme, with two missed cleavages allowed, carbamidomethyl cysteine as the fixed modification; oxidized methionine and acetylation of the N-terminal as the variable mod, 20 ppm mass tolerance on precursor ions, and 0.05 Da on fragment ions. The false discovery rate (FDR) was set at <1% for peptide sequences. RAC1 expression in exosomes was confirmed by western blotting.

#### Proteomics analysis of A549 cells

Proteins were extracted and digested. Peptides were labeled using the amine-reactive TMTsixplex Isobaric Mass Tagging Kit (#90064B, ThermoFisher Scientific) and separated into eight fractions by reversed phase chromatography at pH 12. Peptides were resuspended in 0.1% formic acid and separated by Rigol L3000 HPLC liquid chromatography equipped with a Thermo Acclaim PepMap 100 C18 Trap Column and a Waters BEH C18 Analytical Column (China). Elution was carried out at a flow rate of 600 nL/min, in which solvent A (0.1% formic acid in water) and solvent B (0.1% formic acid in 80% acetonitrile) were applied. The elution gradient was 5-20% B (2 min), 10-40% B (80 min), 40-55% B (2min), 55-90% B (2 min), and 90-100% B (5 min).

The eluate was sprayed into a Q Exactive Orbitrap Mass Spectrometers (ThermoFisher Scientific) at a voltage of 2.5 kV. Full scans ranging from 350 to 1500 m/z were acquired at a resolution of 60,000 (at 200 m/z) with an automatic gain control target value of 3 × 10^6^ and a maximum ion injection time of 20 ms. MS scans were recorded in profile mode, while the MS/MS was recorded in centroid mode. Three replicate injections were performed for each set of samples. Data were processed using Proteome Discoverer 2.2 software (ThermoFisher Scientific). Peptides with scores above 20 and below 0.05 (the significance threshold filter) were selected for analysis. Single peptide identification required a score equal to or above the identity threshold. MS/MS database searches were conducted using the SEQUEST search algorithm within the Proteome Discoverer Tool (ThermoFisher Scientific). The workflow included the spectrum selector, SEQUEST search nodes, and the percolator. Trypsin was specified as the protease, and a maximum of two missed cleavages were allowed. MS and MS/MS mass tolerances were set to 10 ppm and 0.02 Da, respectively. A false discovery rate of 1% was set at the PSM level as well as the protein level. Gene names of encoded proteins identified in the proteomics analysis were uploaded into the online STRING 11.0 database (https://string-db.org) for GO analysis. LC-MS/MS testing was conducted by Novogene Co., Ltd. (China).

### Bioinformatics analysis

The Protein Atlas Database (https://www.proteinatlas.org/) was used to evaluate BCAR1 expression in a variety of cell lines, including LUAD cells. PPI STRING software 11.0 (https://string-db.org/cgi/input.pl) and Cytoscape (https://cytoscape.org/) Programs were used to analyze potential interactions between BCAR1 and RAC1. The Gene Ontology (GO) Program, which is powered by PANTHER (http://geneontology.org/) was used for enrichment analysis of molecular functions.

### Establishment and evaluation of tumor xenograft animal models

#### Construction of tumor xenograft animal models

H1975 cells were injected into BALB/c nude mice subcutaneously and intraperitonealy. H1299 cells were subcutaneously and intraperitonealy injected into NCG/NPG mice due to their relatively poor tumorigenic ability. Approximately 200 µL of cells was subcutaneously injected into the right forearm (2×10^7^ cells/mL) or intraperitonealy injected into the right lower abdomen (1×10^7^ cells/mL). The tumor formation and body weight were observed every 3-4 days before euthanization in 28 days. Tumors and metastases were collected or counted.

#### Detection of CTCs in mice using the PepMNPs method

To detect CTCs in xenografted mice, peripheral blood was collected before euthanization. CTCs were isolated and characterized using iron oxide magnetic nanoparticles conjugated to the EpCAM recognition peptide using PepMNPs method 14,22. DAPI(+)/CK(+)/CD45(-) cells were defined as CTCs.

### Statistical analysis

The comparison of measured data was performed using Student's *t*-test or paired-samples *t*-test for statistical analysis. Pearson correlation was used for continuous variables. The prognostic factors were examined by univariate and multivariate analyses using the Cox proportional hazard model. K-M plots were generated for prognostic survival analysis. All analyses were performed using SPSS 26.0 Software (SPSS, USA). *P* < 0.05 (two-sided) was considered statistically significant.

## Results

### BCAR1 plays critical roles in CTCs by promoting EMT and up-regulating CD274 expression in LUAD

#### High expression of BCAR1 by CTCs which associates with high expression of CD274 and EMT, is a predictor of poor prognosis

**Figure 1:**
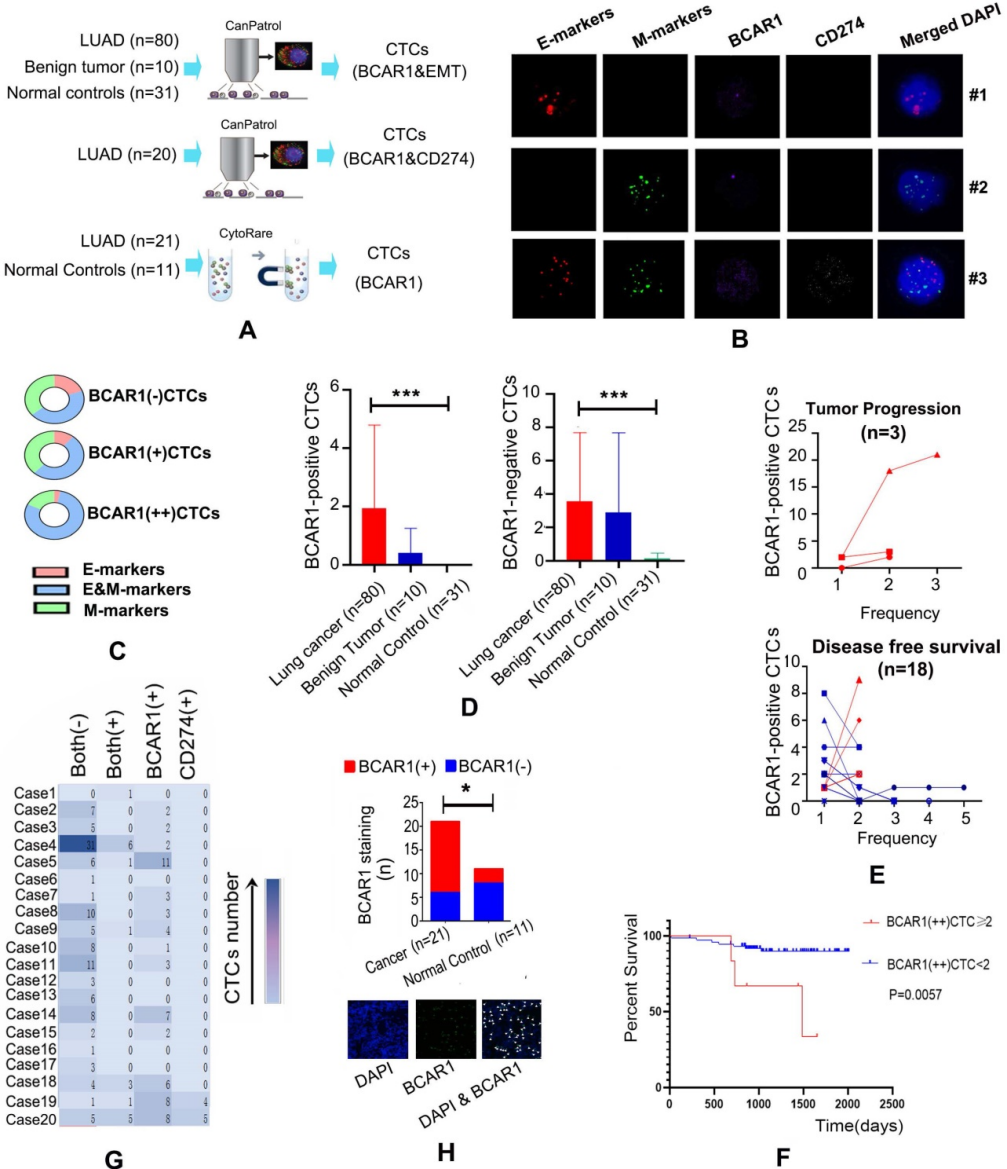
** CD274 expression and EMT contribute to the carcinogenetic role of BCAR1 in CTCs in LUAD. A.** Flow chart of CTCs detection. **B.** Expression of BCAR1 (purple dots), CD274 (white dots), epithelial markers (E-markers, red dots) and mesenchymal markers (M- markers, green dots) as determined by RNA *in situ* hybridization. **C.** Frequency of E&M markers in BCAR1(++)CTCs > in BCAR1(+)CTCs > in BCAR1 negative CTCs (22/28 vs. 64/126 vs.127/285, *P*<0.05). **D.** Compared with controls, BCAR1 negative CTCs and BCAR1 positive CTCs were more abundant in LUAD (*P*<0.05). **E.** Proportion of BCAR1 positive CTCs decreased or remained unchanged in 15 of 18 cases with disease-free survival, and increased in 3 of 18 cases with tumor progression (15/18 vs. 0/3, *P*<0.05). **F.** K-M plotter indicating the presence of BCAR1(++)CTCs prior to treatment was indicative of poor prognosis. **G.** Co-expression of BCAR1 and CD274 (X^2^ test, P<0.001). **H.** BCAR1 expression (green dots) was high in CTCs isolated by the CytoploRare method. Compared with controls, BCAR1 positive cells were more abundant in LUAD (15/21 vs. 3/11, P<0.05).

The strategy used to detect CTCs is shown in Fig. 1A. RNA *in situ* hybridization results indicated that CTCs express BCAR1 (purple dots), CD274 (white dots), E-markers (red dots) and M-markers (green dots) (Fig. 1B). Among the 80 LUAD cases, there were 285 BCAR1 negative CTCs and 154 BCAR1 positive CTCs in the peripheral blood from 67 cases. We classified the 154 BCAR1 positive CTCs into 126 BCAR1(+)CTCs with one purple signal point and 28 BCAR1(++)CTCs with at least two purple signal points. The frequency of E&M markers was as follows: BCAR1(++)CTCs > in BCAR1(+)CTCs > in BCAR1 negative CTCs(22/28 vs. 64/126 vs.127/285, *P*<0.05) (Fig. 1C), indicating that higher expression of BCAR1 was more prone to EMT process. Moreover, only four BCAR1 negative CTCs were detectable in the four healthy controls (4/31), which were remarkably lower compared with LUAD (*P*<0.05) (Fig. 1D). There were few CTCs detected in ten benign tumors, including 22 BCAR1 negative CTCs and 15 BCAR1 positive CTCs (Fig. 1D). LUAD of the left lower lobe showed the highest number of BCAR1 positive CTCs (Fig. S1B). However, there was no significant disparity compared with BCAR1 negative CTCs.

Among the 80 patients, 21 cases underwent dynamic CTCs monitoring (Fig. 1E). There were 18 cases with DFS and three cases with tumor progression. For the cases with DFS, the proportion of BCAR1 positive CTCs decreased or remained unchanged. For the cases with tumor progression, the proportion of BCAR1 positive CTCs increased. Furthermore, the Cox model indicated that BCAR1(++)CTCs prior to treatment were an indicator of poor prognosis (HR=1.705; 95%CI: 1.055-2.756, *P*<0.05), and the K-M plot is shown in Fig. 1F.

As shown in Fig. 1G, there were 20 cases that simultaneously underwent detection of BCAR1 and CD274 expression in CTCs. A total of 18 CTCs with co-expression of BCAR1 and CD274 were found in seven cases. Results of the X^2^ test indicated that co-expression of CD274 and BCAR1 was statistically significant (*P*<0.001).

#### High expression of BCAR1 by CTCs of LUAD is validated by the CytoploRare method

BCAR1 (green dots) were identified by immunofluorescence staining in CTCs isolated by the CytoploRare (Fig. 1H). The proportion of BCAR1 positive CTCs in LUAD was significantly higher than that in controls (15/21 vs. 3/11, *P*<0.05).

### BCAR1 induces CD274 expression and EMT via RAC1 signaling and BRD4 shuttling, respectively

#### BCAR1-KO and BCAR1-OE were established in LUAD cells

BCAR1 expression is high in a variety of cells and cell lines, including endothelial cells from veins and skin, breast cancer cells, and LUAD cells (Fig. S1C). BCAR1 expression is also high in H1975, H1299, and A549 cells (Fig. S1C). These cells were used for the subsequent experiments.

**Figure 2:**
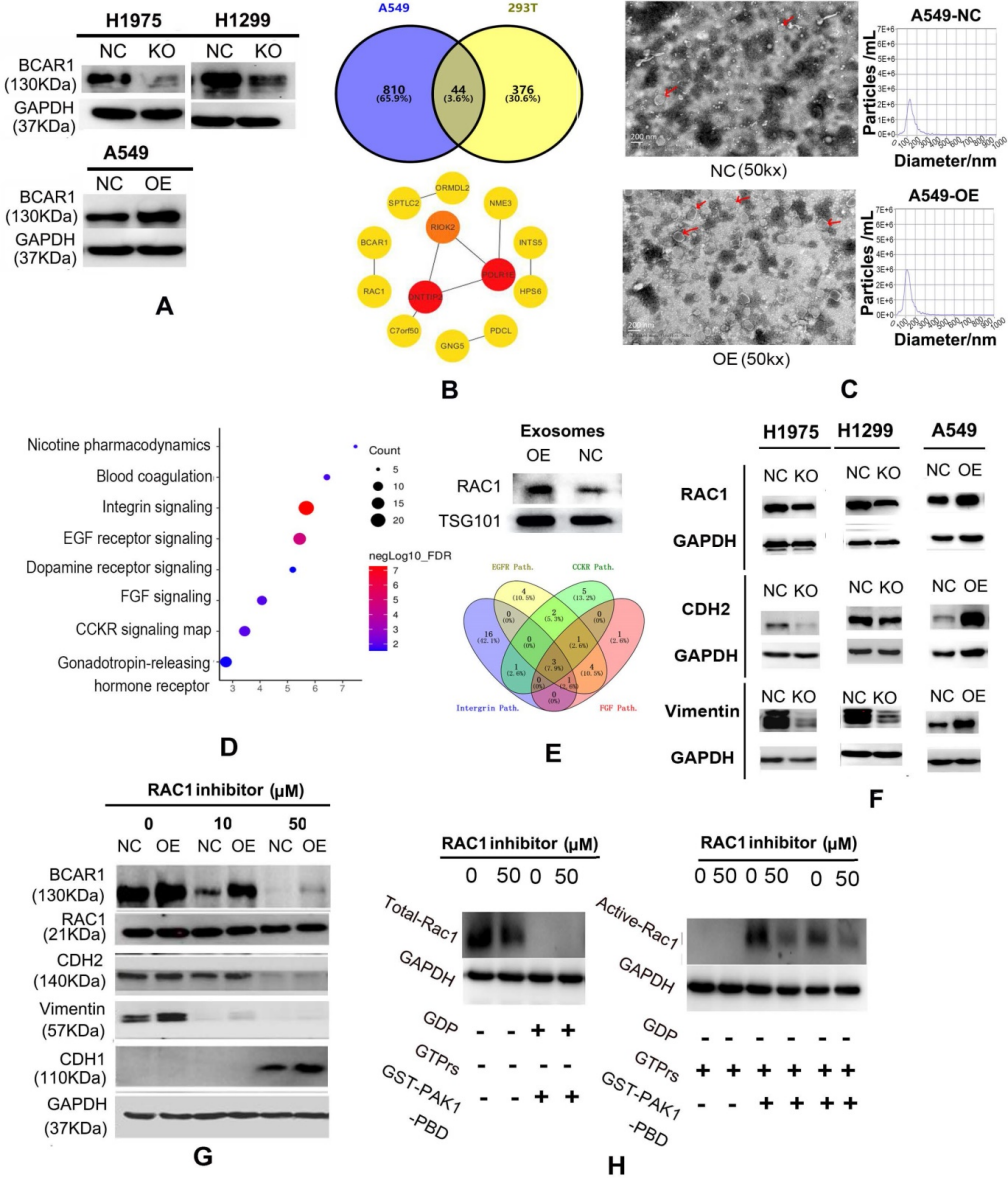
** RAC1 plays a role in the carcinogenesis of cells and exosomes in LUAD. A.** Western blotting results showing successful BCAR1-KO in H1975 and H1299 cells and BCAR1-OE in A549 cells. **B.** Following BCAR1-OE in A549 cells, the expression of 854 genes was significantly up-regulated, as detected by MS. Forty-four proteins overlapped with 420 proteins that were previously identified to interact with BCAR1. **C.** Electron microscopy results and particle size analysis showing exosomes of BCAR1-OE A549 cells were more abundant and significantly smaller than those of controls. **D.** MS results and bioinformatics analysis revealing the signaling pathways that involved 405 over-expressed genes in exosomes following BCAR1-OE. **E.** Carcinogenetic pathways with top four significant FDRs. Integrin, EGFR, CCKR, and FGF had three overlapped genes, i.e., RAC1, GRB2 and CDC42. Western blotting showing increased RAC1 expression following BCAR1-OE. **F.** Expression of RAC1 and M-markers was decreased in H1975 and H1299 cells following BCAR1-KO, whereas it was increased following BCAR1-OE in A549 cells. **G.** Following treatment with NSC-23766 at 50 μM, BCAR1 expression was decreased in BCAR1-OE A549 cells and controls. CDH2 and vimentin levels were also decreased, whereas the CDH1 level was detectable. **H.** Compared with controls, the active RAC1 level was decreased by 41.78% following treatment with NSC-23766.

BCAR1 expression was reduced by 43% and 30% in H1975 and H1299 cells, respectively, following BCAR1-KO (Fig. 2A and Fig. S1D). Results of q-PCR and western blotting revealed successful and significant BCAR1-OE in A549 cells (Fig. 2A, Fig. S1D and Table S9).

#### RAC1 plays important roles in the carcinogenesis of BCAR1 in LUAD cells and exosomes

Following BCAR1-OE in A549 cells, the expression of 854 genes was up-regulated by at least 25%, as revealed by MS. There were 44 proteins (Fig. 2B, Table S10) that overlapped with the 420 potential proteins that may interact with BCAR1 23. The PPI string filtered 13 hub genes and indicated that RAC1 has the only linkage to BCAR1 (Fig. 2B).

Electronic microscopy identified numerous exosomes in the culture media of A549-BCAR1-NC cells and A549-BCAR1-OE cells, as shown in Fig. 2C. However, particle size analysis indicated the exosomes of BCAR1-OE cells were significantly smaller and more abundant than those of BCAR1-NC cells (Fig. 2C and Fig. S1E).

Furthermore, 2333 and 2251 proteins in exosomes of BCAR1-NC cells and BCAR1-OE cells, respectively, were detected by MS. The expression of 405 genes (Table S11) by BCAR1-OE cells was significantly higher by at least a quarter compared with BCAR1-NC cells. Pathway analysis of the 405 genes by GO (Fig. 2D) showed the signaling pathways with the top four FDRs, i.e., integrin (FDR: 5.47E-8), EGFR (FDR: 1.18E-7), CCKR (FDR: 2.03E-5), and FGF (FDR: 1.10E-2).

Three overlapping genes, (RAC1, GRB2 and CDC42), were involved in the four signaling pathways (Fig. 2E). Western blotting results confirmed that BCAR1-OE can up-regulate RAC1 expression in exosomes (Fig. 2E and Table S9).

#### RAC1 is critical for BCAR1 in the induction of EMT in LUAD

The expression of RAC1 and M-markers (CDH2 and Vimentin) was significantly decreased in H1975 and H1299 cells following BCAR1-KO, and increased after BCAR1-OE in A549 cells (Fig. 2F, Fig. S1F, Table S9). However, the expression of an E-marker, CDH1, was undetectable in these cells (data not shown).

Following treatment with NSC-23766, RAC1 expression was slightly reduced (Fig. 2G), whereas active-RAC1 expression was significantly decreased by 41.78% in A549 cells (Fig. 2H). Following treatment with NSC-23766 and inactivation of RAC1, BCAR1 expression was completely inhibited in both A549-BCAR1-NC and A549-BCAR1-OE cells (Fig. 2G), indicating a potential interaction between BCAR1 and RAC1.

**Figure 3:**
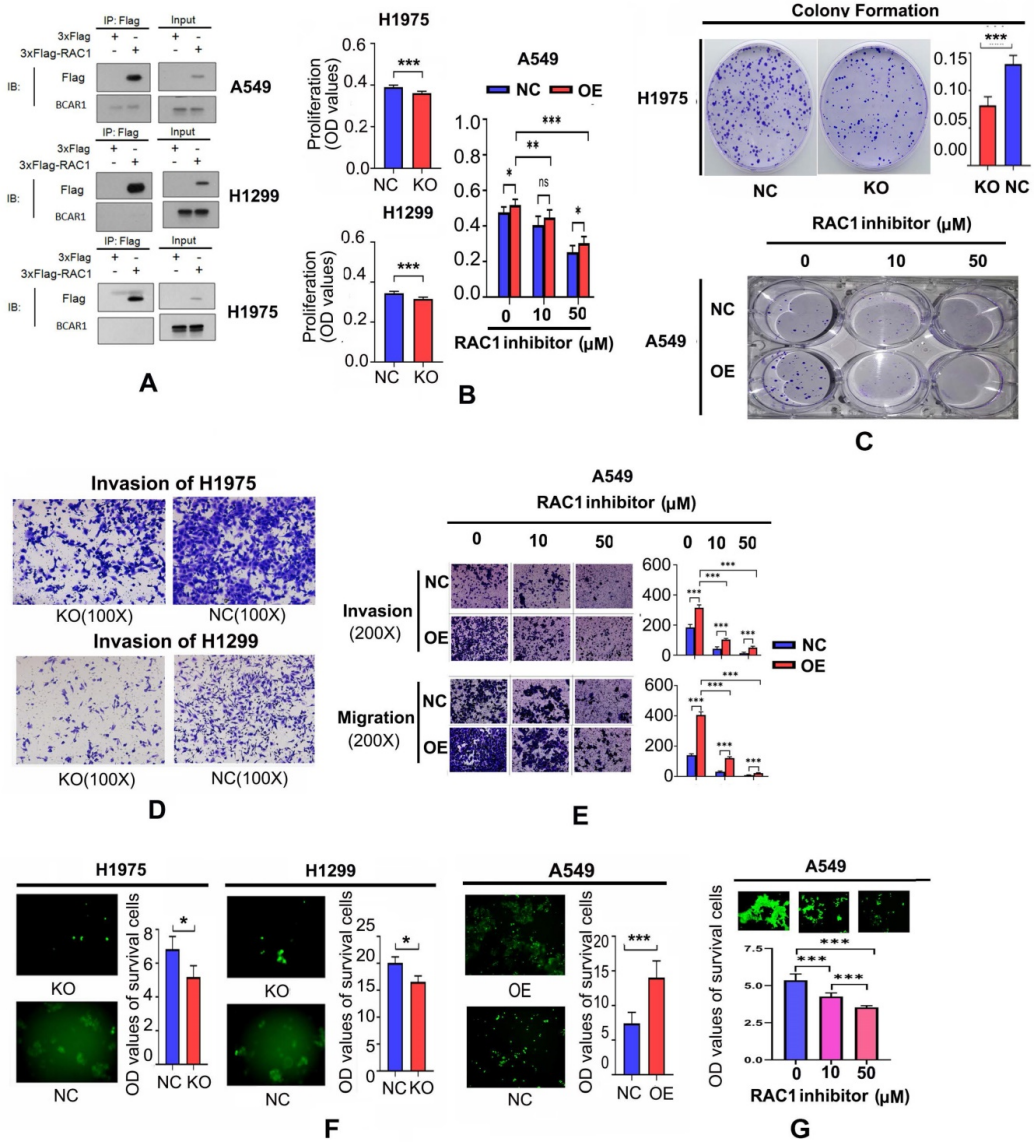
** RAC1 functions with BCAR1 in cell proliferation, colony formation, cell invasion and migration, and resistance to anoikis in LUAD. A.** Co-IP results failed to show a direct interaction between BCAR1 and RAC1 in LUAD cells. **B.** Cell proliferation was inhibited after BCAR1-KO in H1975 and H1299 cells and promoted after BCAR1-OE in A549 cells. BCAR1-NC and BCAR1-OE in A549 cells reduced cell proliferation following treatment with NSC-23766. **C.** Colony-formation was significantly decreased and increased in H1975 and A549 cells following BCAR1-KO and -OE, respectively. Colony-formation of A549-BCAR1-OE cells was significantly antagonized by NSC-23766 treatment. **D.** Cell invasion was decreased after BCAR1-KO in H1975 and H1299 cells. Both invasion and migration were increased following BCAR1-OE in A549 cells. **E.** Enhanced invasion and migration following BCAR1-OE in A549 cells were significantly antagonized following treatment with NSC-23766. **F.** Survival of A549 cells following BCAR1-KO was significantly lower than that of respective controls in H1975 and H1299 cells (*P* < 0.05). Survival of A549 cells following BCAR1-OE was significantly higher than that of the control in A549 cells (*P* < 0.001). **G.** BCAR1 induced anoikis resistance in A549 cells following BCAR1-OE, which was antagonized by NSC-23766 treatment.

Following inhibition of RAC1, the expresion of CDH2 and vimentin was also decreased, whereas CDH1 expression was detectable (Fig. 2G), demonstrating RAC1 is critical for EMT. However, Co-IP results did not show a direct interaction between BCAR1 and RAC1 in LUAD cells (Fig. 3A).

#### RAC1 is critical for BCAR1 to enhance the proliferation, colony formation, invasion, migration, and resistance to anoikis of LUAD cells

As shown in Fig. 3B, cell proliferation was inhibited following BCAR1-KO in H1975 and H1299 cells, and promoted after BCAR1-OE in A549 cells. As shown in Fig. 3C, colony formation was reduced after BCAR1-KO in H1975, and increased after BCAR1-OE in A549 cells. However, there was no difference in this parameter in H1299 cells (data not shown).

Cell proliferation and colony formation of A549-NC and A549-OE cells was inhibited following treatments with NSC-23766 (Fig. 3B,C). As shown in Fig. 3D, cell invasion was remarkably decreased after BCAR1-KO in H1975 and H1299 cells (496.40±45.33 vs. 321.80±56.26, *P*<0.001 and 359.00±39.84 vs. 143.40±10.64, *P*<0.0001). Furthermore, both invasion and migration were increased following BCAR1-OE in A549 cells (247.2±12.28 vs. 415.0±15.51, *P*<0.001 and 69.20±6.64 vs. 109.6±17.14, *P*<0.01). Finally, BCAR1-promoted invasion and migration was significantly antagonized after treatment with NSC-23766 (Fig. 3E).

As shown in Fig. 3F, the OD values of surviving cells decreased or increased after BCAR1-KO or BCAR1-OE. Additionally, BCAR1-induced resistance to anoikis was antagonized by NSC-23766 treatment (Fig. 3G).

#### BCAR1 can up-regulate CD274 expression by shuttling BRD4-S into the nucleus

**Figure 4:**
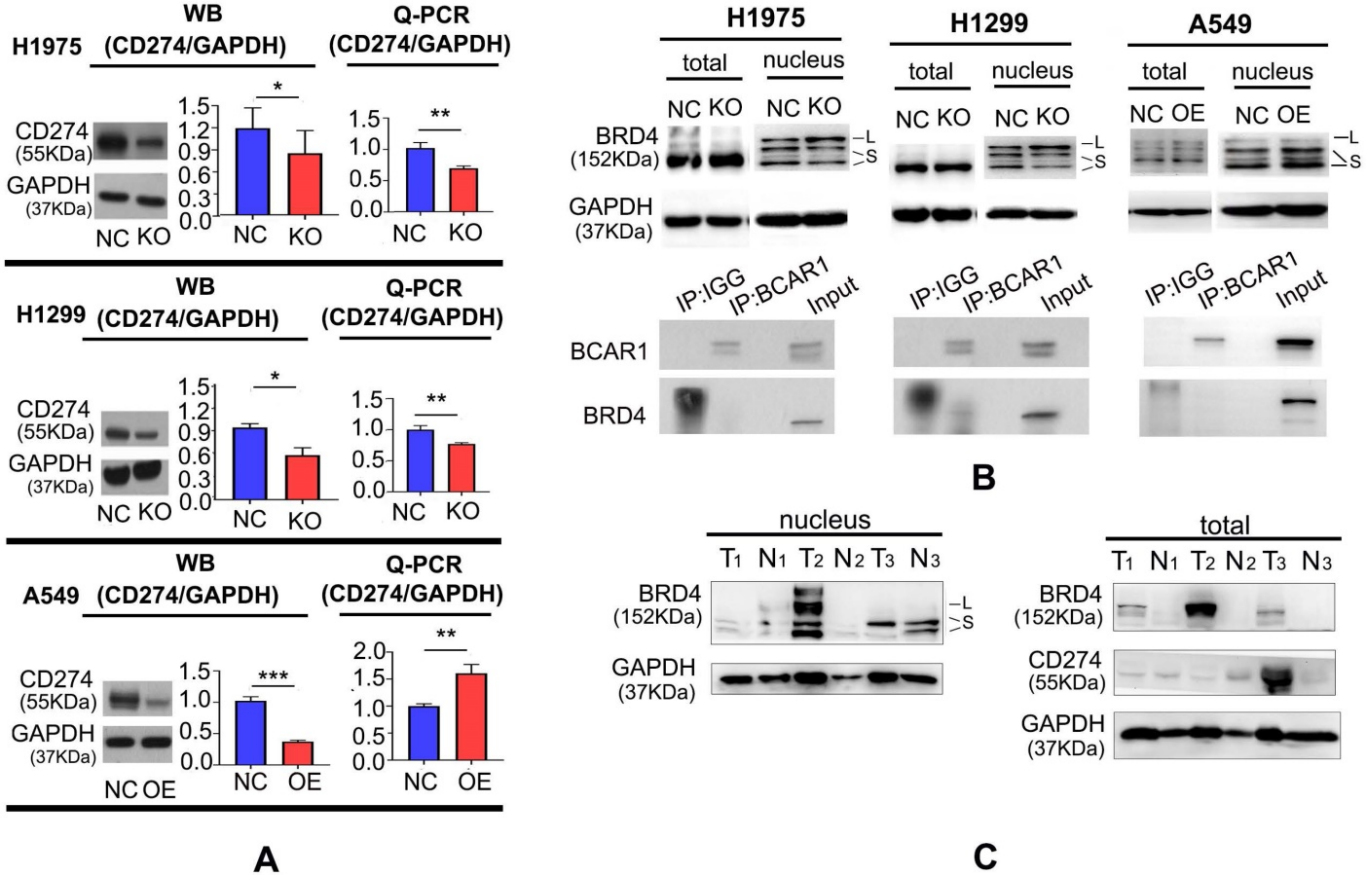
** BCAR1 can up-regulate CD274 expression by shuttling BRD4-S into the nucleus. A.** CD274 expression was decreased following BCAR1-KO in H1975 and H1299 cells and increased following BCAR1-OE in A549, as determined by q-PCR. CD274 expression was decreased following BCAR1-OE in A549 cells, as determined by western blotting. **B.** BRD4 expression in the nucleus can be divided into a long isoform (BRD4-L) and a short isoform (BRD4-S); Expression of BRD4-L and BRD4-S was significantly increased and decreased following BCAR1-KO, respectively, whereas BRD4-S expression was significantly increased following BCAR1-OE in A549 cells. The interaction between BCAR1 and BRD4 in H1299 cells was confirmed by Co-IP. **C.** CD274 expression was high in tumor tissues with BRD4-S expression and without BRD4-L expression (T3). However, CD274 expression was low in tumor tissues with BRD4-L expression and without BRD4-S expression (T2).

As shown in Fig. 4A and Table S9, CD274 expression was decreased following BCAR1-KO in H1975 and H1299, as demonstrated by western blotting and q-PCR, and increased following BCAR1-OE in A549, as indicated by q-PCR. However, CD274 expression was decreased in BCAR1-OE A549 cells, as verified by western blotting.

By immunoprecipitation mass spectrometry (IP-MS), we previously reported that BCAR1 may interact with BRD4 23, which may directly target the transcription of CD274 and lead to the encoding of PD-L1 24. Indeed, the interaction between BCAR1 and BRD4 could be confirmed by Co-IP in H1299 cells (Fig. 4B).

Fig. 4B and Table S9 also showed that BRD4 expression in the nucleus can be divided into a long isoform (BRD4-L) and a short isoform (BRD4-S). The expression of BRD4-L and BRD4-S in the nucleus was increased and decreased, respectively, following BCAR1-KO. By contrast, the expression of BRD4-S in the nucleus, was significantly increased following BCAR1-OE, indicative of the potential carcinogenetic role of BRD4-S and the anti-carcinogenetic role of BRD4-L.

As shown in Fig. 4C and Table S9, high CD274 expression was found in tumor tissues (T3) with BRD4-S expression but without BRD4-L expression. Similarly, the CD274 level was lower in the absence of BRD4-S in the nucleus (T1) or in the presence of high BRD4-L expression in the nucleus (T2). These findings indicate that BRD4-S and BRD4-L in the nucleus may enhance or inhibit CD274 expression, respectively.

### *In vivo* studies confirm the co-expression of BCAR1/RAC1 and BCAR1/CD274, and unveil the roles of BCAR1 in the formation and immunoevasion of CTCs

#### CTCs and tumor formation in nude mice were prohibited by BCAR1-KO of LUAD cells

**Figure 5:**
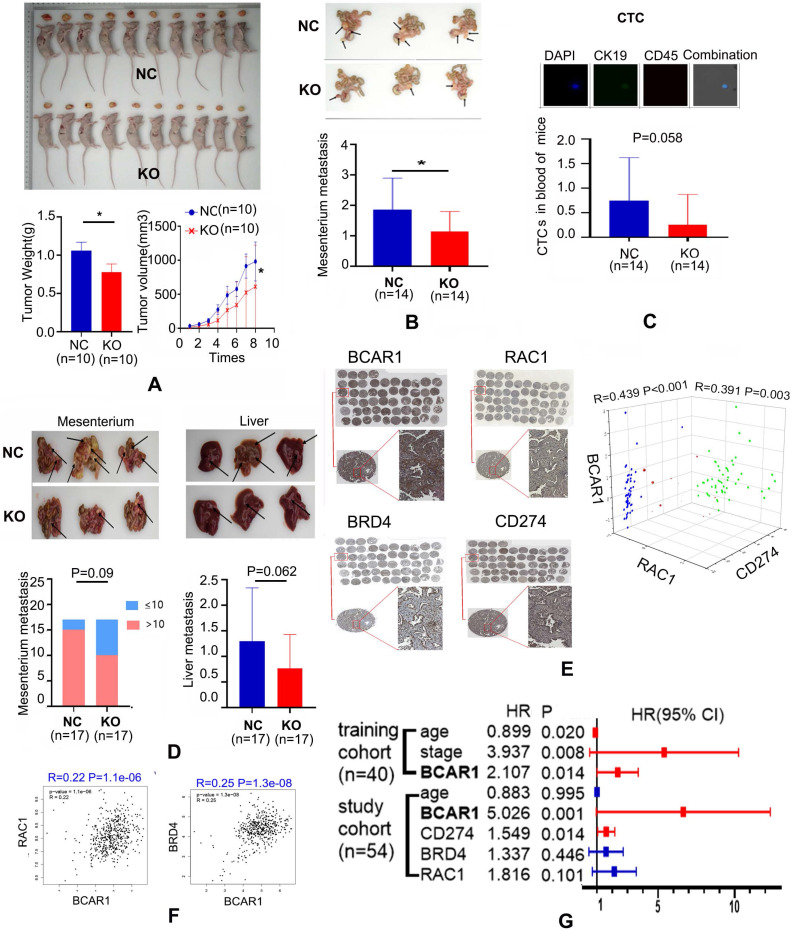
***In vivo* studies confirm the co-expression of BCAR1/RAC1 and BCAR1/CD274 and unveil the role of BCAR1 in CTC formation. A.** Compared with controls, the weight and volume of the subcutaneous xenograft in the H1975-BCAR1-KO group were remarkably lower. **B.** Metastases in the mesenterium by H1975-BCAR1-KO cells were inhibited compared with controls. **C.** Proportion of CTCs in the peripheral blood of the H1975-BCAR1-KO group was significantly lower compared with controls (*P* = 0.058). **D.** Incidence of massive (enormous) metastasis (number >10) in the mesenterium or number of liver metastases showed a decreasing trend (*P* = 0.09 and *P* = 0.062) in the H1299-BCAR1-KO group compared with controls. **E.** IHC results showed the expression of BCAR1, RAC1, CD274, and BRD4 in LUAD tissues. BCAR1 expression was significantly positively correlated with that of RAC1, BRD4, and CD274. **F.** BCAR1 expression was significantly positively correlated with that of RAC1 and BRD4 in LUAD using TCGA data. **G.** High expression of BCAR1 was indicative of a worse prognosis, as validated by training and study cohorts.

Compared with controls, the tumor weight and volume of the subcutaneous xenograft of H1975-BCAR1-KO cells were remarkably lower (Fig. 5A).

**Figure 6:**
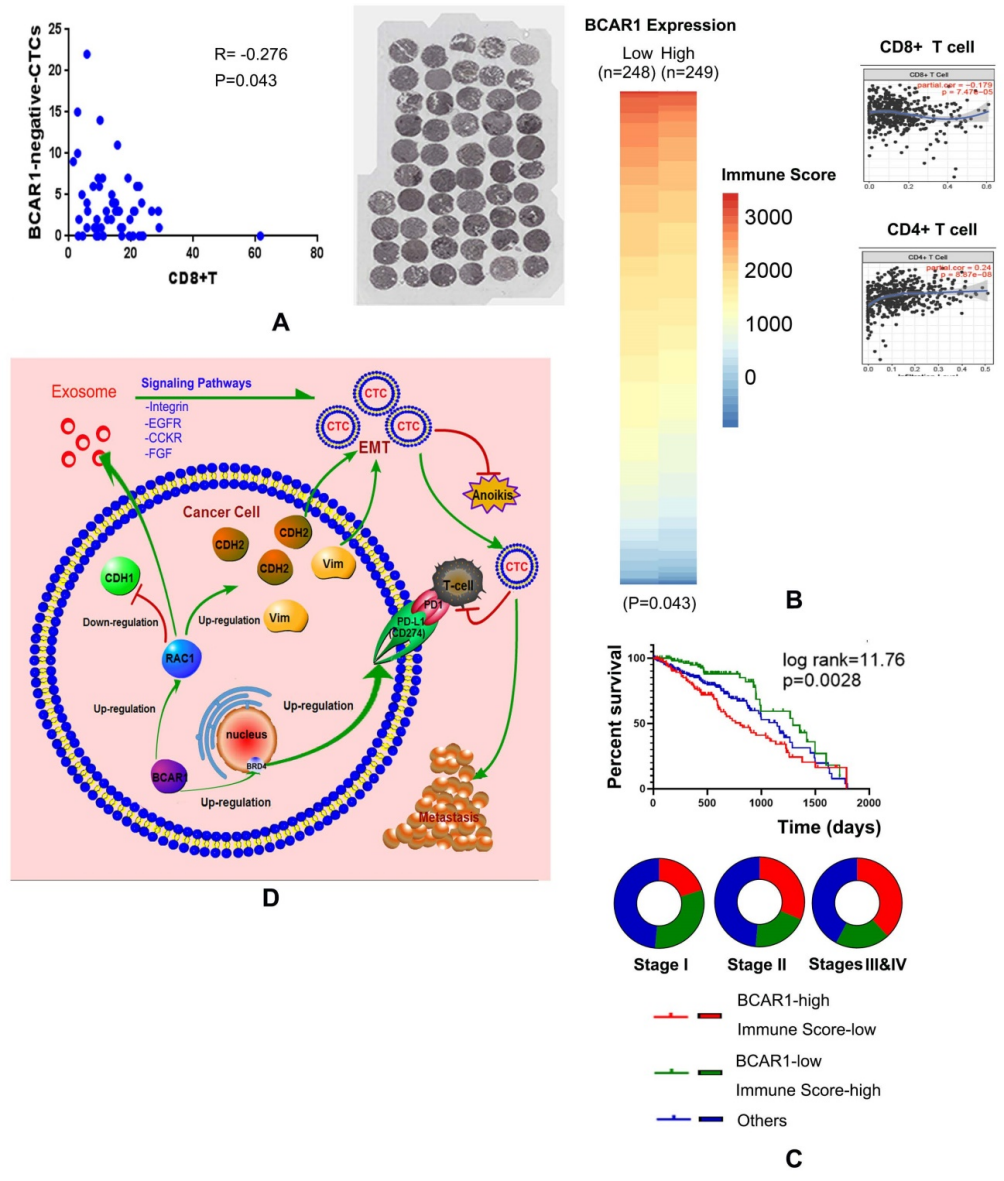
** BCAR1 expression in LUAD tissues predicted poor prognosis, indicating immunoevasion. A.** CD8+T cells were stained and counted. They were negatively correlated with BCAR1(-) CTCs, indicating BCAR1(-) cells in LUAD tissues may be screened and killed by CD8+T cells before entering peripheral blood. **B.** For TCGA data, the heat map indicated that the immune scores of the BCAR1-high group were significantly lower than those of the BCAR1-low group (*P* < 0.05). BCAR1 expression was significantly positively and negatively correlated with CD4+ T cells (P = 8.87×10^-8^) and CD8+ T cells (*P* = 7.47×10^-5^). **C.** K-M plot showing the cases with high BCAR1 expression and a low immune score had the worst prognosis, whereas those with low BCAR1 expression and a high immune score had the best prognosis. The former cases were more common in stages III and IV, and the latter cases were more common in stage I. **D.** Hypothesis on the role of BCAR1 in the formation and immunoevasion of invasive CTCs.

Similarly, metastases of H1975-BCAR1-KO cells in the mesenterium were also suppressed, compared with NC cells (Fig. 5B). Simultaneously, CTCs in the peripheral blood of these mice from the H1975-BCAR1-KO group were marginally significantly lower compared with the NC group, as shown in Fig. 6C (*P*=0.058).

With regard to H1299 cells, the incidence of enormous metastasis (number>10) in the mesenterium or the number of liver metastases showed a decreasing trend with marginal significance (*P*=0.09 and *P*=0.062) in the BCAR1-KO group compared with the BCAR1-NC group (Fig. 5D).

#### Co-expression of BCAR1/RAC1 and BCAR1/CD274 was confirmed in LUAD tissues

Immunohistochemistry results indicated that BCAR1 was expressed in the nucleus and cytoplasm. RAC1 was expressed in the cytoplasm, and CD274/BRD4 were expressed in the cell membrane and nucleus (Fig. 5E). BCAR1 expression was significantly positively correlated with that of RAC1, BRD4, and CD274 (Fig. 5F). The co-expression of BCAR1/RAC1 and BCAR1/BRD4 in LUAD tissues was validated by TCGA data.

#### BCAR1 expression in LUAD tissues predicts poor prognosis and associates with immunoevasion

High expression of BCAR1 predicts a worse prognosis, as validated by the training cohort (OS) and the study cohort (DFS) (Fig. 5G). In the study cohort, CD8+T cells were stained and numbered, and they were negatively correlated with the proportion of BCAR1 negative CTCs (Fig. 6A), rather than with BCAR1 positive CTCs (data not shown), indicating BCAR1 negative cells in LUAD tissues may be screened and killed by CD8+T cells before entering peripheral bloods. These findings indicate that BCAR1 is conducive to tumor evasion.

For TCGA data, 497 LUAD cases were divided into BCAR1-high and -low groups by the medium value of BCAR1 expression. In Fig. 6B, the heat map indicated that the mean immune score of the BCAR1-high group (n=249) was significantly lower than that of the BCAR1-low group (n=248) (*P*<0.05). Furthermore, BCAR1 expression was found to be significantly positively and negatively correlated with the levels of CD4+ T cells (*P*=8.87×10^-8^) and CD8+ T cells (*P*=7.47×10^-5^), respectively.

For TCGA data, 453 cases with OS information were divided into three groups by the medium values of BCAR1 expression and the immune score. The K-M plot (Fig. 6C) indicated that the cases with high BCAR1 expression and a low immune score had the worst prognosis, whereas the cases with low BCAR1 expression and a high immune score had the best prognosis. The former cases were more common in stages III and IV, whereas the latter cases were more common in stage I (Fig. 6C).

## Discussion

Studies focusing on liquid biopsies, including the detection of CTCs are timely and hot. Generally, the detection of CTCs includes enrichment and identification of cells. Enrichment refers to the isolation of CTCs from the peripheral blood, whereas identification includes polymerase chain reaction (PCR), immunofluorescence staining, and RNA *in situ* hybridization experiments 25. Herein, the CanPatrol method was used to capture and identify CTCs based on the amplification of branched DNA signals 26. Both epithelial and mesenchymal markers, as well as other biomarkers, such as BCAR1 and CD274, were simultaneously detected. The CytoploRare method captures CTCs via magnetic beads 27. Both methods have been used in studies of cancer diagnosis, evaluation of cancer recurrence, and chemotherapeutic efficacy 28,29. We used both methods to validate the reliability of BCAR1 expression in CTCs. However, pilot experiments indicated that neither method could capture CTCs in mice (data not shown), because CTCs are smaller in small animals. Therefore, Pep^@^MNPs 14,22, which are iron oxide magnetic nanoparticles, were used to capture and identify CTCs in tumor-bearing mice. Currently, investigators are identifying new CTC biomarkers such as CD274 and EMT markers.

EMT promotes metastasis. In CTCs, EMT confers mesenchymal properties which can promote invasiveness and anoikis resistance 30, 31.

CD274, also known as programmed death ligand 1 (PD-L1), is the principal ligand of programmed death 1 (PD-1). It also controls interactions between tumor-infiltrating T lymphocytes and cancer cells 32. CD274 expression is detected in CTCs 33, where it mediates the immune escape of CTCs 34. However, the roles of CTCs in triggering EMT and upregulating CD274 expression are unclear. This study demonstrated the carcinogenetic roles of BCAR1 in the formation and immunoevasion of CTCs.

High BCAR1 expression in CTCs associates with EMT, and BCAR1 can trigger EMT via RAC1 signaling *in vitro*. RAC1 is widely expressed in cells where it mediates cell remodeling, membrane transport, transcriptional regulation, cell growth, and development 35. A previous study has reported that BCAR1 and RAC1 also regulate cell movement and adhesion 36. Specifically, RAC1-mediated cell migration is initiated by BCAR1 37. Furthermore, the BCAR1/Rac1 axis can suppress colorectal cancer and metastasis through the Hippo, ERK, and PAK signaling pathways 38, indicating the signaling pathway is indispensable in cell signal transduction. In addition, RAC1 regulates EMT 39, potentially via STAT343 40, ERK2 41 and the nuclear translocation of β-catenin 42. Furthermore, RAC1 can inhibit anoikis 43-45. Triclosan-induced EMT by RAC1 activation promotes cell resistance to anoikis 46. Inhibiting the expression and activity of RAC1 can block cell metastasis and restore anoikis 47.

In the tumor microenvironment, exosomes play critical roles in cancer development, metastasis, and immunity 48. Moreover, smaller exosomes render more effective cellular communication between glioma cells and cause faster proliferation and metastasis of cells 49. Intriguingly, RAC1 is enclosed into these exosomes and participates in Integrin, EGFR, CCKR, and FGF signaling pathways in the tumor microenvironment.

There was no direct interaction between BCAR1 and RAC1 in LUAD. It is possible that interactions between these proteins are indirect. Additionally, high expression of BCAR1 in CTCs associates with CD274 expression. BCAR1-KO can decrease CD274 mRNA and protein levels. However, BCAR1-OE only increased the CD274 mRNA level and not protein level. CD274 is found in exosomes in the plasma of tumor patients, where it plays a role in tumor progression 50. We speculate that CD274 is enclosed into exosomes but subsequently degraded (undetectable by MS and western blotting), leading to the disparity between the mRNA and protein levels. This warrants the further study.

There is no reported interaction between BCAR1 and the nucleoprotein, BRD4, the transcriptional promoter of CD274 51 has two isoforms, BRD4-S and BRD4-L. These two isoforms have opposing functions in cancer biology. Numerous studies have demonstrated that BRD4-S has a cancer-promoting effect on the growth and progression of breast cancer, whereas BRD4-L has the opposite effect 52-54. In colon cancer, BRD4-S promotes tumorigenesis 52. Currently, it is unclear which isoform up-regulates CD274 expression. We conclude that BCAR1 mediates the transfer of BRD4 isoforms to the nucleus, and BRD4-S and BRD4-L promote and inhibit CD274 expression, respectively.

Conclusively, as shown in Fig. 6D, BCAR1/RAC1 signaling cascade can trigger EMT, as well as up-regulate the expression of mesenchymal markers (CDH2 and vimentin), and down-regulate the expressions of an epithelial markers (CDH1), causing the formation of CTCs with enhanced properties of invasion, migration, and resistance to anoikis. BCAR1 expression can result in the secretion of more exosomes with high RAC1 expression, which may alter the tumor microenvironment. Moreover, BCAR1 can mediate the transfer of BRD4-S into the nucleus, leading to the up-regulation of CD274 expression in CTCs, which is conducive to the immune evasion.

## Conclusion

BCAR1, as a new target for LUAD treatment, plays carcinogenetic roles in the formation and immunoevasion of invasive CTCs. The mechanism includes triggering EMT via RAC1 signaling and up-regulating CD274 expression by shuttling BRD4-S into the nucleus.

## Supplementary Material

Supplementary figures and table S1-S9.

Supplementary f table S10.

Supplementary f table S11.

## Abbreviations

BCAR1/p130Cas: breast cancer anti-estrogen resistance 1
CTCs: circulating tumor cells
LUAD: lung adenocarcinoma
EMT: epithelial-to-mesenchymal transition
KO: knockout
OE: over expression
OS: overall survival
IHC: immunohistochemistry
EpCAM: epithelial cell adhesion molecule
FDR: false discovery rate
GO: Gene Ontology
IP-MS: immunoprecipitation mass spectrometry
