# Supplementary material for: BCAR1 plays critical roles in the formation and immunoevasion of invasive circulating tumor cells in lung adenocarcinoma
Source: Int J Biol Sci. 2021 Jun 11;17(10):2461–75. doi: 10.7150/ijbs.61790 (PMC8315020; doi:10.7150/ijbs.61790)
Supplement: Supplementary file 1 — Supplementary figures and table S1-S9. [file ijbsv17p2461s1.pdf]

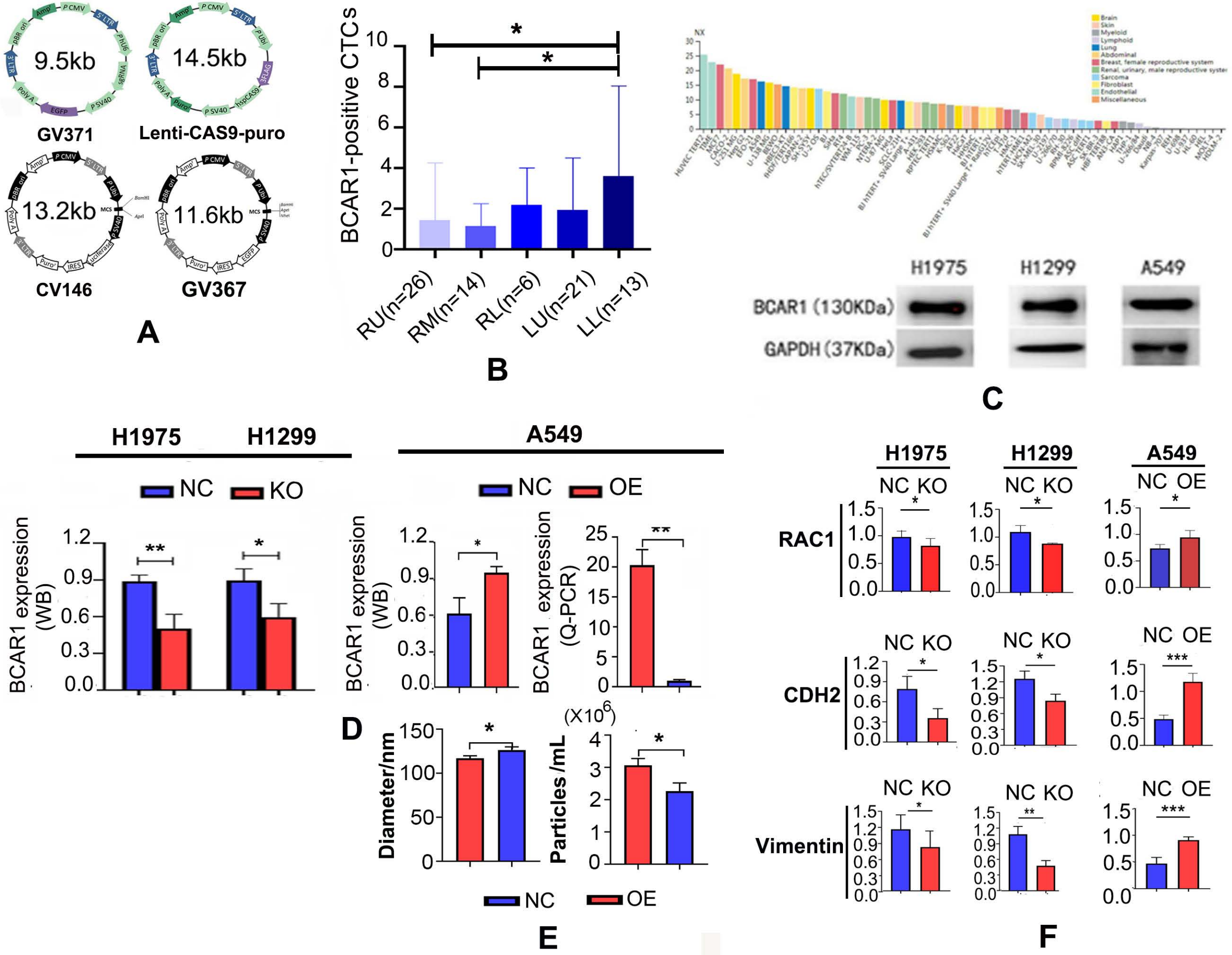

9.5kb
GV371
14.5kb
Lenti-CAS9-puro
13.2kb
CV146
11.6kb
GV367
A
BCAR1-positive CTCs
RU(n=26)
RM(n=14)
RL(n=6)
LU(n=21)
LL(n=13)
B
H1975
H1299
A549
BCAR1 (130KDa)
GAPDH (37KDa)
C
H1975
H1299
NC
KO
BCAR1 expression (WB)
A549
NC
OE
BCAR1 expression (WB)
BCAR1 expression (Q-PCR)
D
Diameter/nm
(X10^6)
Particles /mL
NC
OE
E
H1975
H1299
A549
NC KO
NC OE
RAC1
CDH2
Vimentin
F

Table S1. Clinical and demographical characteristics of training cohort and study cohort

Variables	LUAD tissues		CTCs		
	Training cohort (n=40)	Study cohort (n=54)	Detection by CanPatrol^{TM} (n=80)*	Simultaneous Detection of CD274 (n=20)	Detection by CytoploRare (n=21)
Gender(male: female)	16:24	24:30	39：41	13：7	8:13
Age(year)	58.60±9.46	60.4±9.1	58.10±10.30	64.58±7.51	55.85±13.30
Tumor size(cm)	3.07±1.21	2.3±0.7	1.93±0.91	2.34±1.11	1.93±1.43
Stage(1a:1b:2a)	22:13:5	46:8:0	70：8：2	14：4：2	19:2：0
Survive time(days)	OS:2732.1±880.8	DFS:582.5±385.9	DFS:582.66±375.60	N.A.	N.A.

Note: * 80 cases included 54 cases in Study Cohort.

Table S2. information of antibodies

name	Cat number	company	dilution rate	usage
BCAR1	#13846S	CST	1:200/1：1000/1: 200	IHC/WB/IF
BCAR1	SC-20029	Santa cruz	1:500	IP
CD274	# 13684S	CST	1: 100/1:1000	IHC/WB
BRD4	ab128874	Abcam	1:200/1:500/1:1000	IHC/IP/WB
RAC1	ab33186	Abcam	1:50/1:1000	IHC/WB
CD8	ab17147	Abcam	1:50	IHC
CD4	ab133616	Abcam	1:200	IHC
CDH1	#14472S	CST	1:1000	WB
CDH2	#13116S	CST	1:1000	WB
Vimentin	#5741S	CST	1:1000	WB
GAPDH	#2118	CST	1:1000	WB
TSG101	ab125011	Abcam	1:1000	WB
Flag	F1804	Sigma	1:2000	IP

Table S3. Primers (5'->3') of probes for BCAR1 and CD274 detection in CTC.

ID	seq
BCAR1	cacgtcgtagaggtcaggag;gcatacacaccactgtcgac;cagaaggtgggcaacggtgg;cagatgtgtgggcagcattg; tgtaggtggacgtagtcata;tgttccagtcgttcaaactg;cactgctccaggtagaagag;cggtaaagaaggcgtccacg
CD274	ctactgggaatttgcattca; tagtgcagccaggtctaatt; tcctctccatgcacaaattg; tgtagctactatgctgaacc; gagagctggtccttcaacag; gatctgaagtgcagcatttc; catcctgcaatttcacatct; ctgatcatgcagcggtacac

Table S4. Synthetic oligos for BCAR1 sgRNA construction

NO.	5´	STEM	3´
BCAR1-sgRNA-a	CACCg	CAGCAACGGGTCTCGGCCAT	
BCAR1-sgRNA-b	aaac	ATGGCCGAGACCCGTTGCTG	c
NC		CGCTTCCGCGGCCCGTTCAA	

Table S5. Primers of construction of cDNA library of BCAR1

ID	seq
BCAR1(45429-1)-P1	AGGTCGACTCTAGAGGATCCCGCCACCATGCCTGCCAAGCCCTTCCTCTCTTCTG
BCAR1(45429-1)-P2	CACAGGCTAGCTCAACCGGTTCAGGCGGCTGCCAGCTGGCCTAGGACGCGG

Table S6. Primers of Q-PCR to detect BCAR1 expression in cells

Gene	Upstream primer sequences	downstream primer sequences	Amplified fragment size (bp)
GAPDH	TGACTTCAACAGCGACACCCA	CACCCTGTTGCTGTAGCCAAA	121
BCAR1	CAATGCCTCACTGCTCTTCA	CTCCTTCTGGGTCTTCTCAAAC	247

Table S7. Primers of Q-PCR to detect CD274 expression in cells

Gene	Upstream primer sequences	downstream primer sequences	Amplified fragment size (bp)
GAPDH	TGACTTCAACAGCGACACCCA	CACCCTGTTGCTGTAGCCAAA	121
CD274	ACTGGCATTTGCTGAACG	TCCTCCATTTCCCAATAGAC	148

Table S8 Primers of the construction and identification of FLAG-RAC1 vector

Purpose	ID	seq
Construction of vector	RAC1(63107-6)-p1	GAGGATCCCCGGGTACCGGTCGCCACCATGGACTACAAGGATGACGATG
	RAC1(63107-6)-p2	CACACATTCCACAGGCTAGCTTACAACAGCAGGCATTTTCTCTTC
PCR identification	RAC1(63107-6)-p3	GGGTCAATATGTAATTTTCAGTG
	RAC1(63107-6)-p4	CGTCGCCGTCCAGCTCGACCAG

Table S9 Gray values of WB bands

**Figure2A**	H1975		H1299		A549	
	NC	KO	NC	KO	NC	OE
BCAR1-gray value	90197.588	50284.35	86894.856	47738.463	61985.309	84887.836
GAPDH-gray value	83499.128	84346.16	82677.924	81200.714	87430.501	88206.602
Relative BCAR1	1.0802219	0.596166	1.051004329	0.587906936	0.708966645	0.962375084

**Figure2E**	EXSOMES		
		OE	NC
	RAC1-gray value	65233.019	26003.43
	TSG101-gray value	71831.693	60072.93
	Relative RAC1	0.908137	0.432864

**Figure2F**		H1975		H1299		A549	
		NC	KO	NC	KO	NC	OE
	RAC1-gray value	70275.208	49973.7	74572.48	35744.712	48657.501	76248.534
	GAPDH-gray value	66460.693	60104.14	64833.338	62439.986	53402.643	74803.472
	Relative RAC1	1.0573951	0.831452	1.150218118	0.572465087	0.911144061	1.019318114
	CDH2-gray value	9884.983	5621.184	16120.276	12424.619	8602.326	23759.276
	GAPDH-gray value	14352.912	14109.84	21986.083	21072.548	12760.104	21245.518
	Relative CDH2	0.6887092	0.398387	0.733203636	0.589611612	0.674157985	1.11831945

	Vimentin-gray value	26851.326	10861.26	24154.154	11020.205	8741.912	15779.276
	GAPDH-gray value	18609.548	18468.74	22079.69	21890.962	23194.205	21764.548
	Relative Vimentin	1.442879	0.588089	1.093953493	0.503413464	0.376900696	0.724999021

**Figure4B**		H1975				H1299				A549			
		Total		Nucleus		Total		Nucleus		Total		Nucleus	
		NC	KO	NC	KO	NC	KO	NC	KO	NC	OE	NC	OE
	BRD4-L gray value	0	0	55844.362	70220.238	0	0	52615.754	62904.475	3462.98	3824.507	4692.875	4562.97
	BRD4-S gray value	26595.966	27705.77	80201.747	61082.139	64658.291	50139.661	35124.934	24563.537	17439.54	18493.98	39519.343	44851.115
	GAPDH-gray value	79754.057	52731.14	85803.321	91723.38	69898.279	57636.557	81906.158	85731.25	28029.125	24961.983	43871.309	51971.296

**Figure4C**		Nucleus						Total						
		T1	N1	T2	N2	T3	N3		T1	N1	T2	N2	T3	N3
	BRD4-L gray value	0	0	52533.919	0	0	0	BRD4 gray value	7340.205	4553.79	32823.045	1446.213	9468.296	1084.234
	BRD4-S gray value	1263.96	2529.75	29933.208	0	26549.803	41477.442	CD274 gray value	1115.163	1883.77	1907.82	5141.598	30146.681	6780.962
	GAPDH-gray value	19068.104	18920.3	41212.811	10586.933	24698.761	20609.347	GAPDH-gray value	30498.205	16466.477	28827.861	14410.497	24625.033	34259.225
